# Targeted Next-Generation Sequencing Reveals Clinically Actionable *BRAF* and *ESR1* Mutations in Low-Grade Serous Ovarian Carcinoma

**DOI:** 10.1200/PO.18.00135

**Published:** 2018-11-08

**Authors:** Elizabeth H. Stover, Colleen Feltmate, Ross S. Berkowitz, Neal I. Lindeman, Ursula A. Matulonis, Panagiotis A. Konstantinopoulos

**Affiliations:** **Elizabeth H. Stover**, **Ursula A. Matulonis**, and **Panagiotis A. Konstantinopoulos**, Dana-Farber Cancer Institute, Harvard Medical School; and **Colleen Feltmate**, **Ross S. Berkowitz**, and **Neal I. Lindeman**, Brigham and Women’s Hospital, Harvard Medical School, Boston, MA.

## INTRODUCTION

Low-grade serous ovarian cancer (LGSOC) is a rare (< 5%) subset of epithelial ovarian cancer with unique biologic, clinical, and genetic features.^1^ Compared with those with high-grade SOC (HGSOC), the most common histologic subtype of ovarian cancer, patients with LGSOC are diagnosed at a younger age and have a better prognosis.^2,3^ Standard-of-care treatment of advanced-stage LGSOC and HGSOC is similar: surgical cytoreduction plus platinum and taxane chemotherapy.^4^ However, LGSOC is less responsive to platinum-based chemotherapy compared with HGSOC,^5,6^ possibly because of slower proliferation and fewer abnormalities in the homologous recombination repair pathway (including *BRCA1* or *BRCA2* mutations) in LGSOC.^7^ In contrast, LGSOC may benefit more from endocrine or hormonal therapy (aromatase inhibitors [AIs] or tamoxifen), because a greater proportion of LGSOCs express estrogen and progesterone receptors.^8^ Endocrine therapy is a common treatment of recurrent LGSOC,^9^ and in retrospective studies, it provided benefit as maintenance therapy in the adjuvant setting.^10^ Although recurrent LGSOC can follow a chronic, indolent course, it is incurable with current treatments, and patients often die as a result of their disease, highlighting the need for novel therapies.

We describe two patients with LGSOC whose clinical management was informed by targeted-panel next-generation sequencing (NGS) performed in our institution. The OncoPanel test at Dana-Farber Cancer Institute consists of targeted NGS of formalin-fixed tumor samples covering exons of > 300 cancer-associated genes, plus intronic regions of genes involved in somatic rearrangements.^11-13^ OncoPanel tests report mutations, insertions and deletions, copy number variations, and structural variants. We present clinically relevant alterations identified by OncoPanel in two patients with recurrent LGSOC: a patient with a *BRAF* V600E mutation who derived clinical benefit from BRAF inhibitor vemurafenib, and a patient with progressive disease after durable response to hormonal therapy whose recurrent tumor harbored an *ESR1* mutation associated with resistance to antiestrogen therapy. These cases suggest that patients with recurrent LGSOC may benefit from targeted sequencing to inform selection of targeted agents and, more broadly, to identify rational targeted agents and combinations to treat recurrent disease.

## CASE REPORTS

### Case 1

**Fig 1.**
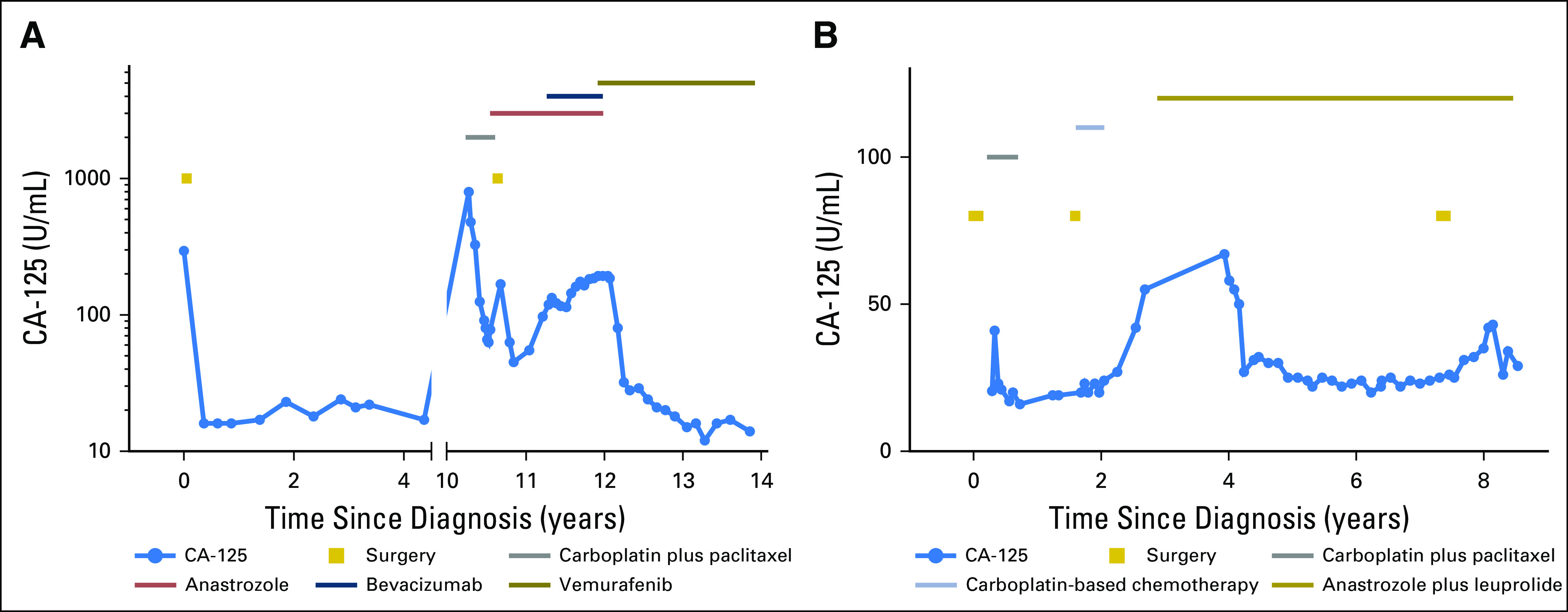
Clinical and treatment histories for (A) case 1 and (B) case 2. CA, cancer antigen.

**Fig 2.**
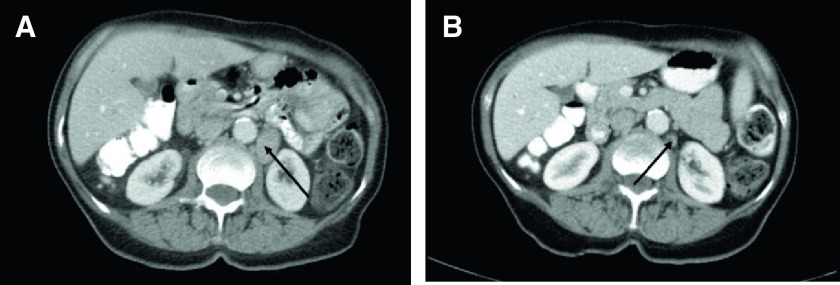
Computed tomography scans for case 1 (A) before (June 2016) and (B) after (May 2017) starting vemurafenib. Arrows indicate decrease in size of lymph node.

The patient was diagnosed at age 61 years with stage IIIB serous borderline tumor, which was surgically resected, with no adjuvant therapy (Fig 1). Ten years later, a computed tomography (CT) scan revealed a mass at the porta hepatis and iliac and periaortic adenopathy; biopsy showed LG serous carcinoma. She underwent carboplatin plus paclitaxel chemotherapy, followed by surgical cytoreduction, with pathology showing LG serous carcinoma. Postoperatively, she received anastrozole, an AI. Subsequently, she developed progressive disease and transitioned to bevacizumab plus anastrozole for 8 months, after which her disease progressed again. On the basis of OncoPanel testing of her tumor from her recurrence surgery, which revealed a *BRAF* c.1799T>A (p.V600E) mutation (Appendix), she started vemurafenib at 480 mg twice daily, which was dose reduced to 240 mg twice daily because of cutaneous toxicity. Her cancer antigen 125 level, which was 193 at initiation of vemurafenib, decreased to 12. A CT scan 1 year after initiation of vemurafenib showed improved retroperitoneal adenopathy and no new sites of disease (Fig 2). As of this report, she continues vemurafenib at 240 mg twice daily (ie, for approximately 20 months) with no new toxicities, no evidence of disease progression, and excellent quality of life.

### Case 2

The patient presented at age 36 years with pelvic pain during in vitro fertilization treatments. A CT scan revealed a fluid-filled adnexal structure, and she underwent surgical exploration. Intraoperative biopsies revealed an incidental serous borderline tumor of the ovary. She underwent complete surgical cytoreduction, with pathology showing stage III invasive serous borderline tumor and one area suggestive for LG serous carcinoma. Two years later, she developed recurrent disease and underwent secondary cytoreduction of metastatic serous carcinoma at multiple sites in the abdomen and pelvis, followed by platinum-based chemotherapy. At subsequent disease progression, she started hormonal therapy with anastrozole, with leuprolide ovarian suppression. During endocrine therapy, she maintained stable low-volume peritoneal disease for almost 5 years (Fig 1). However, she developed a single site of progressive disease (while receiving AI therapy) in an abdominal wall nodule, which was resected. OncoPanel of the recurrent tumor showed an *ESR1* c.1610A>C (p.Y537S) mutation (Appendix). She continued endocrine therapy.

## DISCUSSION

Recent genomic profiling of LGSOC suggested several potential targetable pathways and highlighted dramatic differences in the genomes of LGSOC and HGSOC. HGSOCs have near 100% frequency of *TP53* mutations, few other somatic driver mutations, extensive copy number variations and aneuploidy, and alterations in HRR genes.^14,15^ In contrast, LGSOCs have more stable genomes, with fewer copy number aberrations, but harbor driver mutations in specific oncogenic pathways.^16^ Both serous borderline tumors and LGSOCs have frequent alterations in the RAS/mitogen-activated protein kinase (MAPK) pathway, including mutually exclusive hotspot *KRAS* mutations (G12D and G12V) and *BRAF* mutations (V600E).^17-22^ *NRAS* mutations also cooperate with *EIFAX* to drive proliferation of LGSOC.^23,24^ Whole-exome sequencing of LGSOC shows a low mutation rate and rare recurrent mutations in other genes.^16,22-24^ MAPK pathway mutations in LGSOC led to a clinical trial of the MEK1/2 inhibitor selumetinib, which showed promising activity.^25^ Despite these findings, targeted therapies for LGSOC have not yet been integrated into clinical practice.

We present two cases of LGSOC in which targeted NGS was informative for patient management. The patient in case 1 had recurrent LGSOC and experienced progression throughout chemotherapy, hormonal therapy, and antiangiogenic therapy. Her tumor harbored a *BRAF* V600E mutation. *BRAF* V600E mutations are present in 35% of serous borderline cancers or LGSOCs^18^ and are associated with better prognosis and decreased likelihood of requiring systemic therapy.^18^ Despite the low prevalence of *BRAF* V600E mutations in patients with recurrent disease requiring treatment, this case highlights that this mutation may correlate with sensitivity to BRAF inhibitors, such as vemurafenib. Two previous patient cases of LGSOC with *BRAF* V600E mutations and sustained response to vemurafenib were described.^26,27^ One patient was treated in a basket trial of solid tumors with *BRAF* V600E mutations and responded for at least 12 months.^26^ A second patient, who had experienced progression throughout chemotherapy, hormonal therapy, and antiangiogenic therapy, had a *BRAF* V600E mutation on a recurrence biopsy and was treated with vemurafenib, achieving clinical and radiographic responses.^27^ Despite dose reduction because of skin rash, the patient continued to receive vemurafenib with an ongoing partial response for nearly 2 years.^27^ It is notable that both this patient and our patient seemed to respond to significantly lower doses of vemurafenib than the US Food and Drug Administration–approved dose in melanoma (960 mg orally twice daily), suggesting that LGSOC tumors with *BRAF* V600E mutations may be more sensitive to RAF inhibition. Given that not all solid tumors with *BRAF* V600E mutations respond to RAF inhibition (eg, *BRAF*-mutant colon cancers do not benefit from vemurafenib^28^), these cases illustrate that recurrent LGSOC with a *BRAF* V600E mutation may derive clinical benefit from treatment with vemurafenib or other RAF inhibitors, arguing for routine assessment of these mutations in recurrent LGSOC. Our case further highlights that although skin toxicities can be considerable, dose reduction can be both efficacious and tolerable. In addition to *BRAF* V600E, the high frequency of other alterations in the RAS/MAPK pathway may render LGSOC sensitive to different targeted inhibitors of the pathway. Selumetinib showed a 15% response rate in recurrent LGSOC in a phase II trial.^25^ An exceptional responder in this trial had a response to selumetinib of > 5 years and had a deletion in *MAP2K1* (encoding MEK1), which has oncogenic activity.^22^ Another patient with LGSOC and a *KRAS* G12D mutation had a response of > 7 years to selumetinib.^29^

Our second case of recurrent LGSOC highlights that targeted NGS can elucidate the mechanism of resistance in a patient with excellent response to hormonal therapy and may help tailor future therapy. This patient had a sustained response to hormonal therapy for almost 5 years until she had an isolated recurrent lesion that harbored an *ESR1* Y537S mutation. Although *ESR1* mutations in the ligand-binding domain (most commonly D538G and Y537C/S/N) have been reported in breast cancers treated with hormonal therapy, this is to our knowledge the first *ESR1* mutation reported in LGSOC.^30-35^ *ESR1* mutations are rare in primary, untreated breast cancers but prevalent in metastatic breast cancers resistant to hormonal therapy.^30,33,35,36^ Structural alterations conferred by these activating mutations, including Y537S, result in ligand-independent activation of the estrogen receptor and resistance to endocrine antagonists.^30,36,37^ It is reasonable to surmise that this mutation contributed to tumor progression during AI therapy. Because the remainder of her disease remained stable with anastrozole, the patient continued treatment with the same AI after resection of the resistant lesion. However, in patients who develop progressive disease with *ESR1* mutations, it may be possible to select hormonal agents with alternative mechanisms of action downstream of the activated estrogen receptor and bypass the resistance mechanism. For instance, the oral selective estrogen receptor degrader (SERD) AZD9496 potently binds and downregulates D538G and Y537S ESR1 proteins in vitro and was effective against breast cancer xenografts with Y537S and other *ESR1* mutations, whereas the US Food and Drug Administration–approved SERD fulvestrant had only a partial effect against Y537S.^38,39^ Combinations of antiestrogen agents such as AIs or SERDs with targeted therapies such as mammalian target of rapamycin inhibitors (eg, everolimus) and CDK4/6 inhibitors (eg, palbociclib and abemaciclib) may also overcome resistance associated with *ESR1* mutations. In the PALOMA3 (Palbociclib Ongoing Trials in the Management of Breast Cancer 3) trial of fulvestrant plus palbociclib versus placebo in AI-resistant patients, the benefit of addition of palbociclib to fulvestrant was seen irrespective of specific *ESR1* mutation.^40^ In BOLERO2 (Breast Cancer Trials of Oral Everolimus 2) trial of exemestane plus everolimus, the benefit of everolimus was evident in tumors with an *ESR1* D538G mutation but was not clear in Y537S *ESR1*–mutated tumors because of low numbers.^41,42^ *ESR1* mutations can be detected by targeted sequencing in tumors and cell-free DNA, indicating that clinical testing for these resistance mutations is feasible.^35,36,42^

Tumor heterogeneity is an important challenge in interpreting targetable mutations. In our cases, only a recurrent lesion underwent OncoPanel testing. It is unknown whether each mutation of interest was already present in the primary tumor or acquired in the recurrent tumor. Furthermore, multiple metastatic sites might harbor different driver or resistance mutations; the mutation status of the other residual disease deposits was not tested in these patients. On a practical level, both patients maintained stable low-volume disease with the selected targeted therapy, and identifying different mutations in other lesions might not necessitate a change in management as long as the patients remain asymptomatic.

In conclusion, our first case highlights the potential utility of testing for *BRAF* V600E by targeted sequencing in LGSOC, as well as the possibility of meaningful clinical response to even low doses of vemurafenib in patients with LGSOC with a *BRAF* V600E mutation. In the second case, targeted sequencing helped elucidate the mechanism of resistance in a patient with LGSOC with prolonged response to AI therapy, which could be relevant for selection of additional therapy to overcome resistance. Both cases support that targeted sequencing may be a valuable tool for the clinical management of patients with LGSOC.

## Complete List of Alterations Identified on OncoPanel and Sequencing Metrics

### Description of Test

The OncoPanel assay surveys exonic DNA sequences of 300 cancer genes and 113 introns across 35 genes for rearrangement detection.^11^ DNA is isolated from tissue containing at least 20% tumor and analyzed by massively parallel sequencing using a solution-phase Agilent SureSelect hybrid capture kit (Santa Clara, CA) and an Illumina 2500 sequencer (San Diego, CA). Targeted sequencing is performed on tumor tissue without a matched normal sample; common germline variants are excluded through a series of filtering steps using databases of common single-nucleotide polymorphisms.^11^ All profiling results are interpreted by a molecular pathologist in a formal report.

#### Case 1

##### Metrics.

Estimated 30% tumor; mean of 113 reads; 96% of exons having > 30 reads.

##### Alterations.

BRAF c.1799T>A (p.V600E), exon 15 (12% of 126 reads); ARID1B c.867_867G>GGCA (p.289_289A>AA), exon 1 (36% of 25 reads); CHD1 c.2329G>A (p.D777N), exon 15 (42% of 114 reads); PIM1 c.761T>C (p.F254S), exon 5 (50% of 62 reads).

No somatic rearrangements or copy number alterations were identified, but it was noted that detection of these may have been limited by tumor purity and noise.

#### Case 2

##### Metrics.

Estimated 80% tumor; mean of 363 reads; 98% of exons having > 30 reads.

##### Alterations.

ESR1 c.1610A>C (p.Y537S), exon 10 (in 26% of 303 reads); BCL2L12 c.227_228delGGinsT (p.R76Lfs*23), exon 1 (37% of 132 reads); FLCN c.1022G>A (p.R341Q), exon 9 (29% of 226 reads); FOXL2 c.1025G>A (p.G342D), exon 1 (57% of 138 reads); RAD50 c.2288G>A (p.R763H), exon 14 (45% of 222 reads).

No high-level copy number alterations or somatic rearrangements were detected.
